# Case report: Familial case with autism spectrum and bipolar disorder showing a 20q11.21 microduplication including *TM9SF4*

**DOI:** 10.3389/fpsyt.2023.1240663

**Published:** 2023-11-23

**Authors:** Marly Simoncini, Miriam Violi, Angelo Valetto, Veronica Bertini, Francy Cruz-Sanabria, Leonardo Massoni, Liliana Dell’Osso, Claudia Carmassi

**Affiliations:** ^1^Psychiatric Unit, Department of Clinical and Experimental Medicine, University of Pisa, Pisa, Italy; ^2^Citogenetic Unit, Department of Laboratory Medicine, Azienda Ospedaliero Universitaria Pisana, Pisa, Italy; ^3^Department of Translational Research and of New Surgical and Medical Technologies, University of Pisa, Pisa, Italy

**Keywords:** autism spectrum disorder, mental disorder, bipolar disorder, TM9SF4, microduplication in 20q11.21, neurodevelopment

## Abstract

Autism spectrum disorder (ASD) is characterized by multifactorial etiology and high heritability but can be challenging to be diagnosed, especially in cases presenting subthreshold symptoms with no cognitive or language impairment, which may not be identified until adulthood but may occur in family members of subjects with ASD. This study explores the possible correlation between a genomic imbalance and clinical phenotypes in a family case of a proband with ASD, with subjects presenting full-blown or subthreshold ASD and/or mood disorders. Clinical assessments were carried out by means of the Structured Clinical Interview for DSM-5 (SCID-5) disorders, Autism Spectrum Quotient (AQ), Autism Diagnostic Interview–Revised (ADI-R), Autism Diagnostic Observation Schedule Module 2 (ADOS-2), and Adult Autism Subthreshold Spectrum (AdAS Spectrum). The genetic evaluation included array comparative genomic hybridization (array-CGH). The proband was diagnosed with ASD and bipolar disorder type I (BD-I), her twin brothers with ASD and intellectual disability (ID), and her father and sister with BD type II (BD-II) and autism traits. The proband, her father, twin brothers, and older sister showed a microduplication of 350 kb in 20q11.21. In contrast, the proband’s mother did not present the microduplication or any mental disorder. This study reports a microduplication that segregates with family members affected by ASD or autistic traits comorbid in some cases with bipolar disorder, and that has never been reported in healthy subjects. Among the genes harbored in this region, the *TM9SF4* gene has been recently implicated in risk for ASD.

## 1 Introduction

Autism spectrum disorder (ASD) is a neurodevelopmental disorder (ND) characterized by an impairment of verbal and non-verbal communication, a pattern of narrow interests, and repetitive and stereotyped behaviors (1). The World Health Organization (WHO) estimates the international prevalence of ASD at 0.76% in the general population (1).

Most patients are diagnosed with ASD during childhood and present a mild-to-severe impairment, which is often associated with other neurodevelopmental or mental disorders. Despite increasing attention, many cases, particularly those with normal intelligence, remain undiagnosed or misdiagnosed until adulthood (2). They often come to the attention of the clinician for other mental disorders (such as anxiety, mood disorders, psychoses, trauma, and stress-related disorders) that can mislead the recognition of autistic traits (3–5). For this reason, it is being debated whether some mental disorders could be entangled across the lifespan with autism spectrum subthreshold forms that may constitute a vulnerability factor for mental disorders (6). Furthermore, autistic traits may also be detected in family members of subjects with ASD, either or not comorbid with the same mental disorders, corroborating the role of the interplay among these disorders.

Although the causes of ASD remain largely unexplained, genetic and genomic variations play an important pathogenic role, as highlighted by the high heritability of this disorder (7, 8). A meta-analysis of twin studies estimated heritability in the range of 64–91% (9). In a recent large population-based multinational cohort study, the heritability of ASD was estimated to be approximately 80% (10). ASD is characterized by a remarkable genetic heterogeneity and, so far, more than 100 ASD susceptibility loci have been identified (11). The reported variants range from point mutations to large copy number variants (CNVs) and can be common or rare, *de novo,* or inherited (11). The genes associated with ASD are involved in diverse biological processes, such as synaptic plasticity, chromatin remodeling, gene transcription, and protein degradation (12, 13).

In addition to genetic heterogeneity, literature evidence demonstrates the pleiotropic effect of many genetic factors associated with ASD, where the same genetic alteration may express several phenotypes (11, 14). For example, some genes such as *NFIA1* and *DGL2*, when altered, give rise to ASD and a wide spectrum of neurodevelopmental disorders, strengthening the idea that a single gene can be part of different molecular pathways responsible for different phenotypic outcomes (15, 16).

The complex genotypic and phenotypic architecture of ASD is further complicated by the observation that inherited genomic variants can be present in unaffected siblings and parents (17), reducing the strength of the genotype–phenotype associations. However, considering the complex clinical picture of ASD and the clinical relevance of subthreshold forms of ASD, a thorough phenotypic characterization of relatives of patients is recommended when possible. Subthreshold ASD symptoms, identified in siblings and parents “apparently unaffected” sharing of the same genetic variant present in the full-blown ASD patient, could increase the strength of phenotype–genotype associations.

We report a familial case with five members showing a complex phenotype characterized by either subthreshold or full-blown ASD, along with other neurodevelopmental and psychiatric comorbidities, particularly mood disorders. Array comparative genomic hybridization (array-CGH) analysis highlighted a microduplication of 350 kb in 20q11.21 that segregated all the affected family members. This CNV has never been reported in healthy subjects and harbors several genes, including *TM9SF4*, that have been recently implicated in risk for ASD (18, 19).

## 2 Materials and methods

All participants were clearly informed about the study and had the opportunity to ask questions after having provided written informed consent. All participants were interviewed by means of the Structured Clinical Interview for DSM-5 (SCID-5) (20), a semi-structured interview to diagnose mental disorders according to DSM-5 criteria, and the following self and hetero-administrated tests: Autism Spectrum Quotient (AQ), Autism Diagnostic Interview-Revised (ADI-R), Autism Diagnostic Observation Schedule Module 2 (ADOS-2), and Adult Autism Subthreshold Spectrum (AdAS Spectrum) (21).

### 2.1 Psychiatric questionnaires

- Structured Clinical Interview for DSM-5 (SCID-5) is a *semi-structured* interview that provides a diagnosis based on DSM. The interviewer’s clinical judgment about the interviewee’s responses is required, as well as the interviewer’s knowledge and clinical experience in the field of psychopathology, DSM classifications, and diagnostic criteria (20).
- *Autism Spectrum Quotient (AQ)* is a self-administered tool that measures at what level each adult individual with normal IQ is placed within the continuum of autistic socio-communicative disability, that is, how much each individual presents “traits autistic.” The QA consists of 50 questions, divided into 5 domains, that evaluate 5 different areas as follows: (1) social skills, (2) ability to vary attention, (3) attention to detail, (4) communication, and (5) imaginative ability. For each question, there are 4 possible answers (totally agree, partially agree, partially disagree, and totally disagree). Autistic traits are considered clinically significant total when a score greater than or equal to 32 is reported (21).
- *Autism Diagnostic Interview-Revised* (*ADI-R*) is a semi-structured interview that helps in distinguishing autistic individuals from those with language impairment and mental retardation. It consists of sections on early development, communication, social development and play, repetitive and restricted behaviors, and behavior problems. The ADI-R is divided into three domains as follows: (1) language/communication (cut-off ≥8), (2) reciprocal social interaction (cut-off ≥10), and (3) repetitive behaviors/interests (cut-off ≥3). To receive a diagnosis of autism spectrum disorder, subjects must score over the cut-off points in all domains of the ADI-R (22, 23).
- *Autism Diagnostic Observation Schedule (ADOS)* is a standardized instrument that assesses social interaction, communication, and imagination during a semi-structured interview with a trained examiner. Module 2 (ADOS-2) consists of the following domains: communication (cut-off for autism diagnosis ≥ 3, cut-off for autistic spectrum ≥ 2), social interaction (cut-off for autism diagnosis ≥ 6, cut-off for autistic spectrum ≥ 4), and communication and social interaction (cut-off for autism diagnosis ≥ 10, cut-off for autistic spectrum ≥ 7) (24).
- *Adult Autism Subthreshold Spectrum (AdAS Spectrum)* questionnaire includes 160 items grouped into seven domains, allowing the evaluation of a wider spectrum of manifestations of autism. The responses of the items are binary (yes/no), and domain scores correspond to the sum of positive answers. The “childhood/adolescence” domain includes symptoms related to early developmental phases. The “verbal communication” domain covers features of the speaking questionnaire, including physical contact. The “empathy” domain explores impairment in standing and interpreting facial expressions, intentions, or thoughts, as well as the presence of intense attachment to pets or objects. The “inflexibility and adherence to routine” domain includes difficulty in understanding the subtle aspects of verbal communication, insistence on sameness and habits, unwillingness to eliminate useless objects, and tendency to follow specific procedures. The “restricted interests and rumination” domain includes the tendency to talk about a few preferred topics and being fascinated by numbers and the tendency to waste time over details, to lose track of time, and to take refuge in daydreaming. The “hyper/hypo-reactivity to sensory input” domain explores the tendency to over- or under-react to stimuli, such as textures, smells, noises, temperature, and pain (3).

### 2.2 Molecular analysis

Array comparative genomic hybridization (array-CGH). Genomic DNA of the patients was isolated from peripheral blood by standard methods; DNA from healthy subjects (a male subject and a female subject) was used as control (Agilent Technologies, Santa Clara, California, United States). In total, 200 ng of genomic DNA both from the patient (test sample) and the control (reference sample) were differentially labeled with Cy5-dCTP or with Cy3-dCTP using random primer labeling according to the manufacturer’s protocol (Agilent). The labeling reactions were applied to the 60 K oligo-arrays (Agilent) and incubated for 24 h at 65°C in an oven. Slides were washed and scanned using the Agilent scanner. The identification of individual spots on scanned arrays and quality slide evaluation was performed with the Agilent dedicated software (Feature Extraction, Agilent).

The 60 K slides have a 41 Kb overall median probe spacing (33 KB in Refseq genes).

Copy number variants (CNVs) were identified with Cytogenomics 3.0.6.6 (Agilent), using the Aberration Detection Method 2 (ADM-2) algorithm. This algorithm identifies aberrant intervals in a sample that has consistently high or low log ratios based on their statistical score. ADM-2 uses an iterative procedure to find all genomic intervals with scores above a user/specified statistical threshold value. The threshold was set to a minimum of 6, with the minimum number of probes required in a region of 3 and a minimum absolute log ratio of 0.25. The score represents the deviation of the weighted average of the normalized log ratios from its expected value of zero. It incorporates quality information about each probe measurement.

CNVs classified as pathogenic, likely pathogenic, or variant of unknown significance (VUS) were reported according to the American College of Medical Genetics guidelines and the European Guidelines for constitutional cytogenetic analysis (25, 26).

The CNV detected in the family was reported according to the Genome Reference Consortium Human Build 37 (GRCh37/ hg19).

### 2.3 Bioinformatic analysis

CNV classification was performed using databases such as the Database of Genomic Variants (DGV; see text footnote 1), Database of Chromosome Imbalance and Phenotype in Humans using Ensembl Resources (Decipher),^1^ University of California Santa Cruz (UCSC) Genome Browser,^2^ and Clin Var.^3^

Sfari (see footnote 3), PubMed,^4^ Online Mendelian Inheritance in Man (OMIM) (see text footnote 2) GeneCards,^5^ and Gene Curation Coalition annotations (Gen CC; see text footnote 4) were also checked for evaluating genotype–phenotype association.

The research single-nucleotide variants (SNVs) were performed in the DECIPHER (see footnote 1), ClinVar, Leiden Open Variation, and HGMD databases.

Data on the expression profiles were assessed using GTEx^6^ and the UCSC (see footnote 2).

## 3 Results

**Figure 1:**
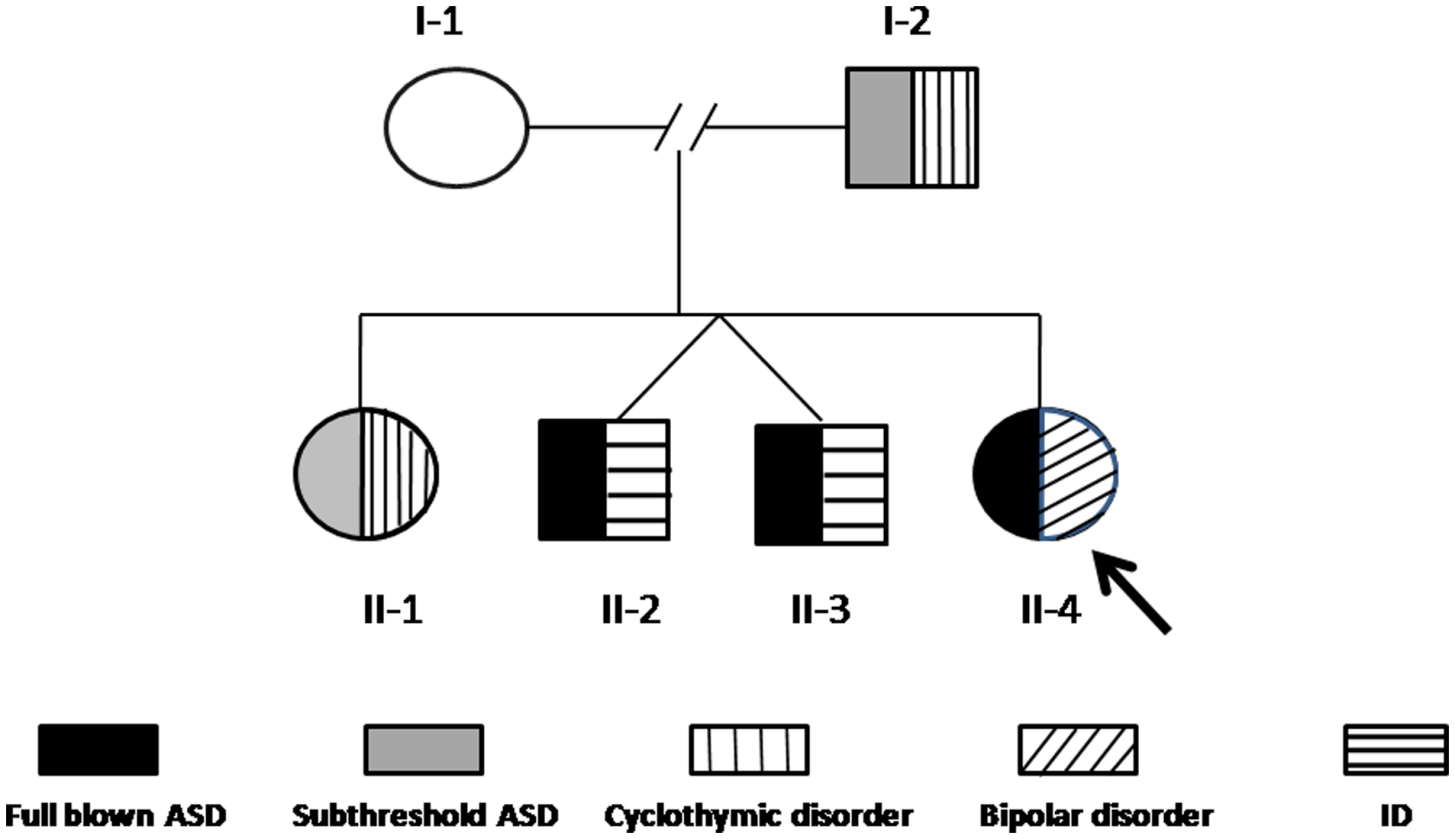
The family tree. The arrow indicates the proband (II-4). The black pattern indicates full-blown ASD, the gray pattern indicates subthreshold ASD, the vertical line pattern indicates BD-II, the horizontal line pattern indicates ID, and the slanting line pattern indicates bipolar disorder I.

Case II-4 is a 24-year-old woman, who was first evaluated at the Psychiatry Clinic of the University of Pisa (Pisa, Italy) for symptoms related to a major depressive episode occurring in the framework of a diagnosis of bipolar disorder I (BD-I), according to the SCID-5. At the time of the first assessment, she reported in her family history two twin brothers (II-2 and II-3) with a diagnosis of ASD and intellectual disability (ID) at an early age. Family members were asked to be assessed by means of psychometric evaluations and genetic analyses included in the present study. In Figure 1, the family tree is depicted. Below are described the clinical features of each family member and the results of the genetic analyses.

### 3.1 Case presentation

Case II-4, the proband, is a 24-year-old woman who received, during childhood, a diagnosis of specific language impairment (SLI), hearing loss, physical abnormality of the speech apparatus, ASD, and apraxia. She required a support teacher at school and was able to obtain basic levels of education. The patient maintained sufficient functioning with discrete relationships with peers and family members. She reported suffering from episodes of bullying during adolescence, which caused subjective distress characterized by emotional lability, crying fits, anxiety, episodes of verbal and physical behavioral dyscontrol in the family contest, and difficulties in social relationships that worsened at the age of 19 after her parent’s divorce. She first received psychiatric treatment at the age of 22 years because of a progressive worsening of mood symptoms with frequent mood swings. She was followed at the local outpatient psychiatric services and administered mood stabilizers (oxcarbazepine up to 300 mg/day) with clinical benefit. The following year, she reported a progressive worsening of depressive symptoms with increasing mood swings, episodes of verbal and behavioral dyscontrol, and increasing suicidal thoughts, which determined her first admission to the inpatient unit of the Psychiatric Clinic of the University of Pisa (Pisa, Italy). Based on the psychiatric evaluation according to the SCID-5, the patient received a diagnosis of bipolar disorder type I (BD-I) in comorbidity with ASD and was treated with risperidone up to 3 mg/day, lithium sulfate up to 124.5 mg/day, chlorpromazine up to 150 mg/day, gabapentin up to 900 mg/day, oxcarbazepine 900 mg/day. Clinical evaluation through structured questionnaires revealed significant ASD symptoms, as measured through both the AdAS (total score = 100/160, cut-off point < 70), the AQ (total score = 40/50, cut-off point < 26), the ADI-R (language/communication: 10; reciprocal social interactions: 10; and repetitive behaviors/interests: 5), and the ADOS-2 (communication: 4; social interaction: 6; communication, and social interaction: 10). During the interview, the patient was described as collaborative, communication was well represented in the elicited and spontaneous component, with a tendency to give answers that were not consistent with the context and a tendency to focus on topics of her interest. She seemed uncomfortable engaging in imaginary games independently of the examiner. She was interested in objects’ details and expressed narrow interests, especially in history and archeology (Figure 1).

Cases II-2 and II-3 are proband’s monozygotic twin brothers, aged 30 years old. They had been followed by the Infant Neuropsychiatry Unit of the University of Pisa (Pisa, Italy) since their childhood and were diagnosed with ASD and ID according to the SCID-5, characterized by impairments in language, intelligence, and motor skills. During childhood, speaking ability was lacking, and there was little attachment to parental figures, especially in relation to separation from parents; there were difficulties in relationships with peers, with a tendency to isolation. The relationship with objects was characterized by a few elementary and stereotyped actions; moreover, it was reported the typical difficulty in imitation games. Over the years, cognitive deficits persisted with difficulties in language that were accompanied by symptoms of anxiety, with a tendency to routine and inflexibility, together with the presence of obsessive control rituals. Both patients reached basic levels of education (middle school) with great difficulty that required continuous support.

Case II-1 is a 33-year-old woman, the sister of case II-4. Her psychomotor development is reported as normal. She describes herself as a sociable person with high energy levels and an irritable temperament. During her childhood, she did not have a good academic performance. She reported a failure at the age of 12 years old, but she was able to obtain a higher school degree. Over the years, the patient developed mood instability with periods characterized by fluctuating energy levels and sporadic episodes of mood bending alternating with episodes in which she felt particularly irritable and energetic with little need for sleep. However, she did not seek medical attention. When she was 30 years old, she underwent psychopharmacological therapy based on SSRI antidepressants and benzodiazepines due to a clinical symptomatology characterized by depressed mood with anxiety. Based on the psychiatric evaluation according to the SCID-5, the patient received a diagnosis of bipolar disorder type II (BD-II). During the clinical evaluation, she was quite cooperative. From the interview conducted through the AQ, the ADI-R, and the ADOS-2, autistic traits emerged, above all, the presence of a particular inflexibility and routine with difficulty in changing daily habits, the conducting of nuanced rituals before starting to write or knocking on the door, the need to proceed in the workplace according to very specific schemes and procedures. She got angry if everything did not go as she wanted and remained rigid about her beliefs, hardly changing a decision already made or an opinion that she considered well founded. There was also reported a tendency to ruminate during the day and even before falling asleep. During the interview, it was observed that the patient had difficulty in grasping the abstract or metaphorical meaning of certain phrases or expressions.

Case I-2 is the proband’s 55-year-old father (also a father of cases II-1, II-2, and II-3). Employed as a workman, he reported a normal psychomotor development with poor academic performances across school ages, along with difficulties in relationships with peers during adolescence and being a victim of several episodes of bullying by peers. He describes himself as a very shy, generous person, not inclined to easily engage in social relationships. He reported a major depressive episode in the aftermath of a stressful vital event (separation from his wife), with depressed mood and emotional lability, frequent crying, feelings of loneliness, and suicidal ideation that ended in a suicide attempt. For this reason, he was treated with psychopharmacological therapy for approximately 1 year, following discontinuation of psychiatric treatments and full recovery that persisted over the years. Based on the psychiatric evaluation according to the SCID-5, he received a diagnosis of BD-II with lifetime depressive episodes, often underdiagnosed and undertreated. The clinical evaluation through structures questionnaires revealed that the patient showed significant ASD symptoms, as measured through the AQ (total score = 32/50, cut point < 26), the ADI-R (language/communication: 9; reciprocal social interactions: 10; and repetitive behaviors/interests: 4), and the ADOS-2 (communication: 2; social interaction: 4; and communication and social interaction: 7), with high scores in the routine and inflexibility domains that suggested the patient’s tendency to be particularly habitual, precise, orderly, and inflexible. Moreover, the patient reported high scores in the childhood and adolescence domains with difficulty in relationships with peers, restricted interests at school, a tendency to clinophilia, fear of socializing on recreational occasions, and social phobia (specific anxieties and phobic features, interpersonal sensitivity). During the interview, the patient was quite cooperative. Speech was mainly represented in the elicited component, less in the spontaneous one. Eye contact was elusive. Mimic and gesticulation were underrepresented. He seemed uncomfortable engaging in imaginary games, regardless of the examiner.

Case I-1 is the proband’s 50-year-old mother (also a mother of cases II-1, II-2, and II-3). She did not have any neurodevelopment problems, and she worked as a clerk in a private company. The reports showed good work functioning. She is a smiling, sociable, optimistic person. Despite the family difficulties, she shows herself as a strong and well-groomed woman. According to the tests performed (AdAS, AQ, ADI-R, and ADOS-2) and clinical interview, she did not satisfy the criteria for any mental disorder (Table 1).

**Table 1:** Clinical questionnaire scores.

Family member	I-1	I-2	II-1	II-4
Age	50	55	33	24
Sex	Female	Male	Female	Female
**Clinical questionnaires**	**Direct score**	**Direct score**	**Direct score**	**Direct score**
**AdAS Spectrum**				
(I) Childhood/adolescence domain	3/21	10/21	4/21	14/21
(II) Verbal communication domain	2/21	8/18	6/18	13/18
(III) Non-verbal communication domain	4/21	8/28	5/28	13/28
(IV) Empathy domain	0/21	5/12	3/12	5/12
(V) Inflexibility and adherence to routine domain	10/43	12/43	12/43	27/43
(VI) Restrictive interests and rumination domain	0/21	8/21	7/21	19/21
(VII) Hyper-Hyporeactivity to sensory input domain	0/21	5/17	3/17	9/17
AdAS spectrum total score	19/160	56/160	40/160	**100/160** ^*^
**AQ**				
Social skills domain	0/10	14/10	1/10	6/10
Attention switching domain	2/10	3/10	7/10	3/10
Attention to detail domain	0/10	7/10	5/10	15/10
Communication domain	3/10	2/10	0/10	10/10
Imagination domain	0/10	5/10	3/10	6/10
AQ total score	5/10	**32/50** ^*^	16/10	**40/50** ^*^

^*^Score above the threshold.

### 3.2 Genetic analysis

Array-CGH detected a CNV that segregates the affected members of this family (I-2, II-1, II-2, II-3, and II-4), and it is not present in the mother (Figure 1). This CNV is a duplication of approximately 350 Kb in 20q11.2, starting from position 30,712,111 and ending at position 31,062,545 (GRCh37/hg19).

This duplication has never been reported in healthy subjects (DGV).^7^ According to the RefSeq genes database, it harbors eight genes. Three of them, *POFUT1*(*607491), *KIF3B* (*603754) and *ASXL1*(*612992) are also reported in OMIM.^8^ Details about the biological role of genes included in this CNV are summarized in Table 2.

**Table 2:** Genes harbored in the duplicated region.

Gene	Function	Omim disease	Inheritance	Phenotype
*TM9SF4* (transmembrane 9 superfamily member 4)	Involved in several processes, including positive regulation of protein localization; regulation of intracellular pH; and vacuolar proton-transporting V-type ATPase complex assembly			
*TSPY26P* (*Homo sapiens* testis specific protein, Y-linked 26)	PSEUDOGENE			
	Predicted to enable chromatin binding activity and histone binding activity. Predicted to be involved in nucleosome assembly			
*PLAGL2* (PLAG1 like zinc finger 2)	Involved in the recognition of DNA and/or RNA by a zinc-finger domain			
*POFUT1* (protein O-fucosyltransferase 1)	Adds O-fucose residues to epidermal growth factor-like repeats of a number of cell surfaces and secreted proteins. O-fucose glycans are involved in ligand-induced receptor signaling.	Dowling-Degos disease 2	AD	characterized by reticular pigmentation
		(MIM #615327)		
*MIR1825*	Non-coding RNA			
*KIF3B* (kinesin family member 3B)	Involved as a heterodimer with kinesin family member 3A to aid in chromosome movement during mitosis and meiosis.	Retinitis pigmentosa 89 (RP89) (MIM #618955)	AD	characterized by classic features of RP as well as features of ciliopathy, including postaxial polydactyly and renal and hepatic disease
	Involved in the intracellular movement of membranous organelles.			
*ASXL1* (ASXL transcriptional regulator 1)	Similar to Drosophila, an additional sex combs gene, which encodes a chromatin-binding protein, is required for normal determination of segment identity in the developing embryo.	Bohring-Opitz syndrome (MIM #605039)	AD	(MIM #605039) characterized by severe intrauterine growth retardation, poor feeding, profound mental retardation, trigonocephaly, prominent metopic suture, exophthalmos, nevus flammeus of the face, upslanting palpebral fissures, hirsutism, flexion of the elbows and wrists with deviation of the wrists and metacarpophalangeal joints
	Thought to disrupt chromatin in localized areas, enhancing the transcription of certain genes while repressing the transcription of other genes.			
	Involved in co-activation for the retinoic acid receptor in cooperation with nuclear receptor coactivator 1.	Myelodysplastic syndrome, somatic (MIM #614286)		
	Mutations in this gene are associated with myelodysplastic syndromes and chronic myelomonocytic leukemia.			
*NOL4L* (nucleolar protein 4-like)	*Homo sapiens* chromosome 20 open reading frame 112			

**Figure 2:**
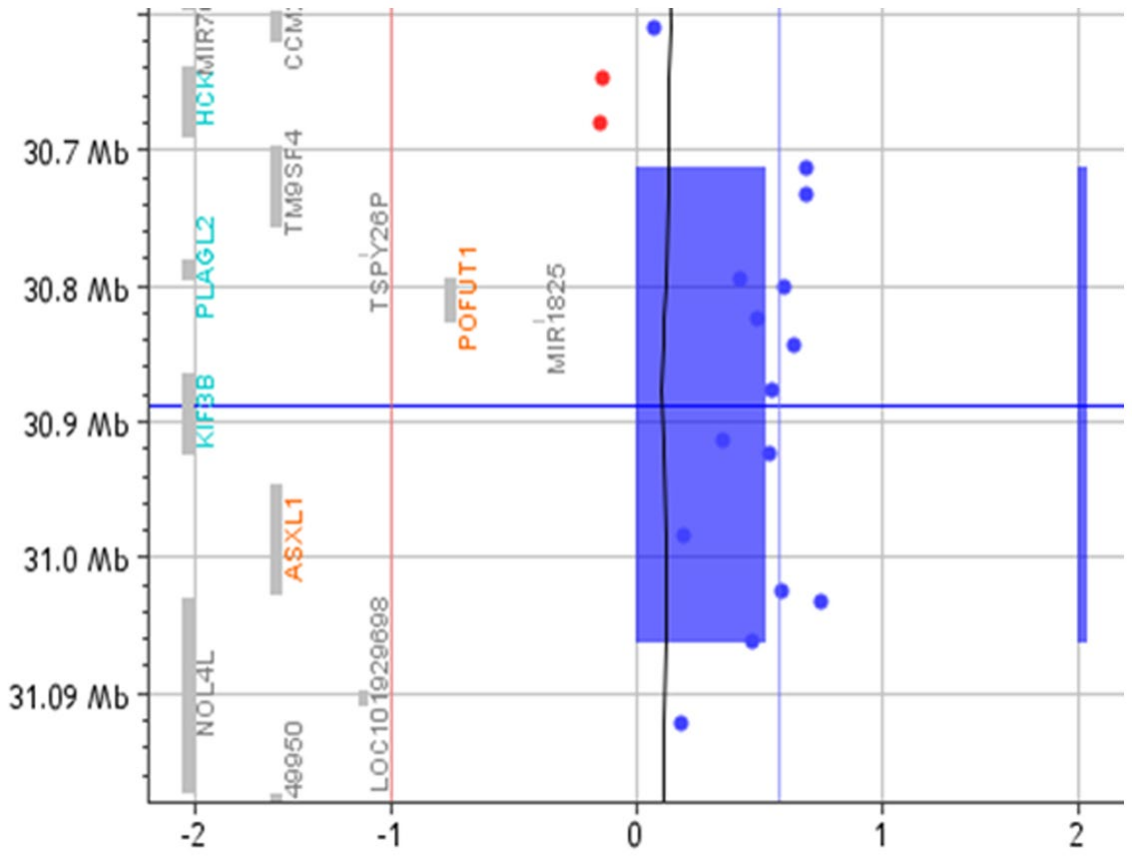
Array-CGH with the 20q11.21 duplicated region. The red vertical line represents the threshold for deleted probes (−1). In contrast, the vertical blue line represents the value of 0.58, where the duplicated probes are expected to fall. The blue rectangle and the blue bar highlight the duplicated region.

A search for the potential link between these genes and ASD highlighted that *TM9SF4* is reported in Simons Foundation Autism Research Initiative (SFARI),^9^ a database for the autism research community with up-to-date information on causative and candidate genes for ASD. *TM9SF4* presents a “sfari gene score” of 1 and an “eagle score” of 0.5. Moreover, according to the Gene Curation Coalition annotations (Gen CC),^10^ which provide information on gene–disease relationships based on online resources and on diagnostic laboratories that share their internal curated gene-level knowledge, this gene could be related to ASD (Table 2; Figure 2).

Genetic counseling was performed on the proband and her family, where the recurrence risk and the implications of this duplication were discussed.

## 4 Discussion

We discuss a family case report with five members characterized by the presence of either subthreshold or full-blown ASD measured through AdAS and AQ questionnaires, along with other neurodevelopmental and psychiatric comorbidities. The proband (II-4) was diagnosed with ASD and BD I; her father (I-2) showed significant ASD symptoms along with BD-II; her sister (II-1) full-filled criteria for the same disease, and although she reported ASD symptoms on the AdAS questionnaire, those did not reach cut-off points for a diagnosis of ASD; her twin brothers (II-2 and II-3) had been diagnosed with ASD along with ID. The mother (I-1) was the only member of the family who did not report current or lifetime mental disorders, as confirmed by the psychiatric assessments.

ASD is mainly diagnosed during childhood in subjects with impaired social functioning, verbal and non-verbal communication, and narrow interests, often associated with mental, speech, and psychomotor delay. These clinical features are all present in the twin patients (II-2 and II-3) who received an early full-blown ASD diagnosis. Instead, subjects with mild forms of autism, with normal or above-average intelligence, may be misdiagnosed or diagnosed only in adulthood when they come to clinical observation for other late-onset psychiatric disorders in comorbidity with ASD (3, 27). Accordingly, the proband (II-4) came to our attention for BD-I, and the familial anamnesis revealed a psychiatric disorder (BD-II) in her father I-2. Despite having encountered some difficulties during their training, both of them were able to obtain a high school degree. Case I-2 was able to engage in work activities, whereas Case II-4 presented poorer relational, social, and occupational functioning. Both showed mild autistic features and, according to the AQ test, were diagnosed with ASD at the ages of 23 and 52 years, respectively.

Previous studies reported that 57% of young patients with bipolar disorder, 38% with major depressive disorder, and 25% with anxiety disorder exceeded the clinical cut-off for quantitative ASD scales. Thus, supra-threshold and subthreshold autistic traits can be considered a vulnerability factor for different types of psychiatric disorders (28, 29), and there is a strong correlation between ASD and catatonia spectrum (30). Some characteristics associated with autistic psychopathology (such as brooding thinking, social withdrawal, rigidity, perfectionism, social phobia, anhedonia, and lack of empathy) could be reconsidered as an autistic core shared by different types of disorders, reflecting the high comorbidity rates between ASDs and other mental disorders (31).

Case II-1 did not meet the cut-points for a diagnosis of ASD but has clinically assessed subthreshold traits of the disorder and particular personality traits discussed above. This is in agreement with the literature showing that female subjects seem to maintain better socio-social skills than male subjects, especially if they have high-functioning autism. Some non-social traits, in fact, appear less severe in adult females who display less eccentric and peculiar interests (32) or less frequent and pervasive stereotyped activities than in males (33). Women with subthreshold autism may develop the ability to camouflage or hide their social insecurities (34), and this may explain the less marked autistic traits in this patient and normal social skills.

The family reported here, with many affected members, is an excellent candidate for the research of genetic alterations. The array-CGH is the first-tier test to be used in the case of AD, ASD, ID, and DD since it has been shown to have a great detection rate in this population (25). This analysis highlighted a duplication of 350 kb in 20q11.2 that segregates in all the affected members (I-2, II-1, II-2, II-3, and II-4), whereas it was not present in the unaffected mother (I-1).

This CNV has never been reported in healthy subjects. To evaluate its clinical significance, we looked at the available public databases that collect individuals with CNVs and pathological phenotypes. No overlapping duplications are present in Decipher, Clin Var, and Copy Number Variation Morbidity Map of Developmental Delay; all the cases reported here show either larger imbalances or additional alterations elsewhere in the genome. Only five “pure” 20q11.2 duplications are reported in the literature, but all of them are much larger and span several megabases (35). These cases show a quite severe clinical picture, including metopic ridging/trigonocephaly, developmental delay, epicanthal folds, and short hands, that could be related to genes not harbored in the duplication described here. In these cases, ASD or mood disorders were rarely reported; this can be explained by the fact that in the databases, clinical features are not fully reported or that psychiatric tests have not always been administered.

As far as the gene content is concerned, this duplication encompasses the following eight genes: six coding protein genes (*TM9SF4, PLAGL2, POFUT1, KIF3B, ASXL1, and NOL4L*), one pseudogene (*TSPY26*), and one non-coding RNA (*MIR1825*; Table 2). Heterozygous loss of function of *POFUT1, KIF3B,* and *ASXL1* is causative of syndromes (#615327, #618955, and #605039; Table 2), apparently not related to the clinical features of this family. However, the phenotypic effects of the duplication of these genes have never been described. Few data are available about *PLAGL2* and *NOL4L* (Table 2); thus, it cannot be excluded that their duplication may contribute to the phenotypic picture.

*TM9SF4* is ubiquitously expressed and highly present in the central nervous system (CNS). Interestingly, its variants have been recently identified in studies of extremely large cohorts of ASD cases. A *de novo* missense variant predicted to be pathogenetic was identified in an ASD proband from the Autism Sequencing Consortium, while four protein-truncating variants in this gene were observed in case samples from the Danish iPSYCH study (18, 19). More recently, four additional variants in *TM9SF4* (three missenses and one synonymous) were identified by Zhou et al. in a cohort of 42,607 ASD cases (19).

These findings highlight a correlation between *TM9SF4* and ASD; according to the frequency of these variants in case–control studies and to their predicted effect on the protein functionality, *TM9FS4* is a candidate for ASD with a false discovery rate (FDR) between 0.01 and 0.05 (18).

Even if the presence of other genetic alteration(s) cannot be excluded, the segregation of this CNV, including *TM9FS4,* in the affected members of this family suggests the involvement of this gene in the risk for ASD.

In the literature, a careful description of the clinical pictures related to *TM9SF4* alteration is missing (18, 19); even if it cannot be excluded that other genes in the region could contribute to the phenotype, this is the first report of a family where the clinical outcome of *TM9SF4* is fully evaluated in all the members. In accordance with other genes related to ASD, alterations of *TM9SF4* give rise to a wide spectrum of psychiatric and ND disorders, ranging from mild to severe.

The identification of the same genetic alteration underlying the different clinical pictures of these family members supports the hypothesis that ASD may be considered as a trans-nosographic dimension that may not only represent the starting point for the development of different psychopathological trajectories but also could underlie non-psychopathological personality traits. These different trajectories might be determined by multiple factors, including genetic background, epigenetic modifications, interactions with the environment, and lifetime events (36–40). It should also be considered that exogenous factors, such as the divorce of the parents and the suicide attempt of the father, could have represented strong emotional and behavioral challenges in this family. The identification of genetic/genomic alterations underlying ASD may allow not only a comprehension of the neurobiology of ASD but also an earlier diagnosis. The precious genetic identification of ASD at-risk patients could contribute to early interventions to improve symptom treatments and reduce the risk of developing a full-blown psychiatric disorder.

## 5 Conclusion

We report on a family with five members showing a complex phenotype characterized by either subthreshold or full-blown ASD, along with other neurodevelopmental and psychiatric comorbidities and a duplication of 350 kb in 20q11.2. Even if it cannot be excluded that affected members carry other genetic alterations not detected by this platform, this microduplication segregates with all the affected members of this family, has never been reported in healthy subjects, and harbors several genes, including *TM9SF4* that has been recently implicated in risk for ASD (18, 19). According to these findings, it may play a role in the phenotype. Reports of additional patients with this imbalance, along with functional studies *in vivo* and *in vitro* models, may be useful to confirm the pathogenicity of this microduplication. Our study could contribute to a better understanding of the neurobiology of ASD, even in the subthreshold form and in comorbidity with psychiatric disorders. It might contribute to a more precise inference of the clinical prognosis, including the impact of complex comorbidities, allowing to improve symptoms treatment.

## Data availability statement

The raw data supporting the conclusions of this article will be made available by the authors, without undue reservation.

## Ethics statement

The research was conducted ethically in accordance with the World Medical Association Declaration of Helsinki. The studies were conducted in accordance with the local legislation and institutional requirements. The participants provided their written informed consent to participate in this study. Written informed consent was obtained from the individual(s) for the publication of any potentially identifiable images or data included in this article.

## Author contributions

MS, MV, AV, VB, and CC: conceptualization. MS, AV, VB, and CC: methodology and project administration. AV and VB: formal analysis. MS, MV, AV, VB, and LM: investigation. MV, AV, VB, FC-S, and LM: data curation. MV, FC-S, AV, and VB: writing—original draft preparation. LM, MS, VB, and CC: writing—review and editing. MS, CC, and LD: supervision. All authors contributed to the article and approved the submitted version.

## Footnotes

^1^ https://www.deciphergenomics.org/

^2^ https://genome.ucsc.edu/

^3^ https://www.ncbi.nlm.nih.gov/clinvar/

^4^ http://www.ncbi.nlm.nih.gov/pubmed

^5^ https://www.genecards.org

^6^ https://www.gtexportal.org/

^7^ http://projects.tcag.ca/variation

^8^ http://www.omim.org

^9^ https://gene.sfari.org

^10^ https://thegencc.org/
